# A Case Report on Pseudo-Internuclear Ophthalmoplegia: A Rare Manifestation of Myasthenia Gravis

**DOI:** 10.7759/cureus.46788

**Published:** 2023-10-10

**Authors:** K.V.P. Munasinghe, W.A.D.J.J. Herath, F.H.D.S. Silva

**Affiliations:** 1 General Medicine, Colombo South Teaching Hospital, Colombo, LKA; 2 Geriatrics, Colombo South Teaching Hospital, Colombo, LKA; 3 Internal Medicine, University of Sri Jayewardenepura, Colombo, LKA

**Keywords:** thymoma, acetylcholine receptor antibody, oculomotor nerve palsy, myasthenia gravis, pseudo-internuclear ophthalmoplegia

## Abstract

A 41-year-old male with recently diagnosed diabetes mellitus type 2 presented with drooping of the left eyelid with double vision and was found to have an adduction deficit in the left eye and nystagmus in the abducting right eye during conjugated gaze: a left-sided internuclear ophthalmoplegia (INO). A medial longitudinal fasciculus (MLF) lesion was excluded exhaustively with brain imaging. The possibility of a pseudo-INO was considered. The autoantibody profile demonstrated positivity to acetylcholine receptor (AChR) antibody. Repetitive nerve stimulation (RNS) and electromyography (EMG) were unremarkable. An acetylcholinesterase inhibitor trial showed a significant improvement in the ocular symptoms. Hence, the diagnosis of ocular myasthenia was confirmed. There was no evidence of a thymic hyperplasia. Herein, we discuss pseudo-INO being an atypical presentation of myasthenia gravis (MG), emphasizing the importance of having a strong suspicion despite unremarkable test results.

## Introduction

Myasthenia gravis (MG) is caused by an antibody-mediated immunological response directed against acetylcholine receptors at the postsynaptic membrane of the neuromuscular junction leading to fluctuating fatigability and skeletal muscle weakness [1,2]. It is a relatively uncommon disorder with an annual incidence of approximately 7-23 new cases per million [3]. MG demonstrates a bimodal distribution of age and sex with the first peak occurring during the second to third decades with more female prevalence and the second peak occurring during the sixth to eighth decades with male predominance. It is a treatable disease that exhibits episodic symptoms during the early course of the disease. The manifestations could be more persistent toward the latter part of the disease [4].

MG manifests principally as ocular symptoms for 50% of the presentation. Approximately 15% of all patients with MG have isolated ocular MG as the only manifestation of their disease [5]. There are numerous atypical presentations and mimickers of MG. Despite the early onset, the diagnosis and treatment initiation could be delayed.

Internuclear ophthalmoplegia (INO) represents a focal central nervous system (CNS) lesion in the medial longitudinal fasciculus manifesting as an adductor deficit with dissociated nystagmus on the abducting eye on the lateral gaze [6]. In pseudo-INO, a similar pattern of ocular manifestations presents in the absence of a CNS lesion. We hereby present a case of pseudo-INO as a rare manifestation of MG.

## Case presentation

A 41-year-old male presented with left-sided ptosis and diplopia in all directions with left mild periorbital swelling for five days duration. He had uncomplicated diabetes mellitus type 2 for six months duration. There was no impairment of visual acuity. Furthermore, there were no similar episodes in the past or history of recurrent headaches, head trauma, or insect bites. He did not have constitutional symptoms including fever or symptoms of respiratory tract or hypothyroidism. There was no fatigability. His glycemic control was satisfactory with oral medication. The patient was a nonsmoker and an occasional alcohol consumer.

There was a left-sided oculomotor nerve palsy and left-sided internuclear ophthalmoplegia. The pupils were spared. There was no ascending or descending muscle or bulbar weakness. The rest of the cranial nerve examination and cerebellar, sensory, and motor examination as well as other systems examination were insignificant. He did not have any skin rashes or other features of autoimmune or vasculitis. He did not give a past history of SARS-CoV-2 viral infection and was fully vaccinated against it by the time of presentation.

**Figure 1:**
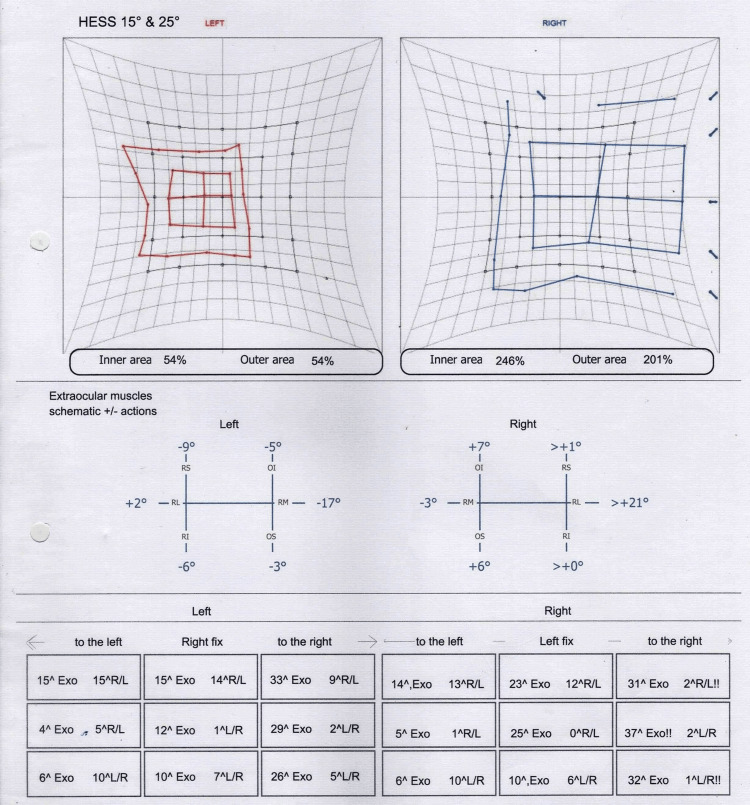
Orthoptic assessment demonstrating incomplete left oculomotor nerve palsy

Basic hematological investigations such as full blood count (FBC), erythrocyte sedimentation rate (ESR), and thyroid function tests (TFTs) were normal. Magnetic resonance imaging (MRI) of the brain detailing the posterior cranial fossa, orbits, and paranasal sinuses was unremarkable. An orthoptic assessment (Figure 1) showed bilateral visual acuity as 6/6 and an incomplete left oculomotor nerve palsy. Computed tomography cerebral angiogram ruled out the presence of a posterior communicating artery (PCOM) aneurysm.

The absence of a genuine INO led to the consideration of pseudo-INO. The etiology for such presentation includes myasthenia gravis, Lambert-Eaton myasthenic syndrome (LEMS) (a paraneoplastic manifestation), or Miller-Fisher syndrome (a Guillain-Barré syndrome (GBS) variant). Repetitive nerve stimulation (RNS) test showed no decremental or incremental (as occurring in LEMS) pattern. The acetylcholine receptor (AChR) antibody level was positive at 0.7 nmol/L (normal: ≥0.5 nmol/L), while the anti-muscle-specific kinase (anti-MuSK) antibody level was within the normal range. A coincidental thymoma was excluded with a negative chest X-ray and contrast-enhanced computed tomography (CECT) of the chest (Table 1). A trial of pyridostigmine 30 mg eight hourly was commenced for one week, which was increased to 60 mg eight hourly for another month. The patient remarkably improved symptomatically, confirming the diagnosis of ocular myasthenia.

**Table 1:** Laboratory parameters ESR: erythrocyte sedimentation rate, TSH: thyroid-stimulating hormone, anti-MuSK: anti-muscle-specific kinase, NCCT: non-contrast computed tomography, MRI: magnetic resonance imaging, CT: computed tomography, PCOM: posterior communicating artery, CECT: contrast-enhanced computed tomography

Investigation type	Investigation results	Reference value
White blood cells	7.36 × 10^9^/L	4-11 × 10^9^/L
Neutrophils	56%	40%-60%
Lymphocytes	38%	20%-40%
Monocytes	3%	2%-8%
Hemoglobin	15.4 g/dL	13-17 g/dL
Platelets	269 × 10^9^/L	150-400 × 10^9^/L
ESR	5 mm/hour	<20 mm/hour
TSH	0.8 mIU/L	0.4-4.0 mIU/L
Acetylcholine receptor antibodies	0.7 nmol/L	Negative: *≤*0.4 nmol/L, borderline: 0.4-0.5 nmol/L, positive: >0.5 nmol/L
Anti-MuSK antibody	0.01 nmol/L	<0.05 nmol/L
Brain NCCT	Unremarkable	
Brain MRI	Unremarkable	
CT cerebral angiogram	Normal CT angiogram, no evidence of a PCOM aneurysm	
Repetitive nerve stimulation test	No decremental potentials	
Chest CECT	No evidence of thymic enlargement or thymoma, normal chest CECT	

## Discussion

INO refers to an abnormal conjugate horizontal gaze characterized by the failure of adduction in one eye and nystagmus in the abducting eye [6]. The term pseudo-INO denotes presentation in the absence of a CNS lesion. The possible mechanism of a "pseudo-INO" could be explained by poor coordination between cranial nerves III, IV, and VI along with their interneuronal pathways due to neuromuscular junction dysfunction due to autoantibody-mediated disruption. Serological evidence plays an important role in the diagnosis. The three main autoantibodies important in pathogenesis are anti-AChR antibodies, anti-MuSK antibodies, and low-density lipoprotein receptor-related protein-4 (LRP4) antibodies. The sensitivity of anti-AChR and anti-MuSK antibodies in ocular myasthenia is 55% and less than 10%, respectively. AChR antibody positivity is associated with thymoma and thymic hyperplasia in more than 75% of cases [7,8]. AChR autoantibody positivity is related to asymmetric ocular symptoms, whereas anti-MuSK antibody positivity demonstrates a symmetrical eye involvement [9]. Decremental pattern in repetitive nerve stimulation observed in myasthenia gravis is not always specific as it is also seen in other conditions such as LEMS, botulism, and motor neuron disease. Single fiber electromyography impairment in neuromuscular transmission shows more sensitivity (80%-95%) than repetitive nerve stimulation (<50%) in the diagnosis of ocular MG [10].

INO occurs mainly due to a lesion in the medial longitudinal fasciculus (MLF), and multiple sclerosis or a stroke could be contributing. There was no history of relapsing-remitting disease in our patient with an accumulation of residual disability confirmed by the absence of hyperintense lesions in the MRI of the brain disseminated in time and space. Therefore, multiple sclerosis remained unlikely. Furthermore, a negative MRI cannot completely rule out multiple sclerosis; thus, the clinical progression of the disease and imaging need to be followed up [11]. He did not give a history of ischemic or hemorrhagic stroke with face, arm, or leg weakness, slurring of speech, or acute-onset visual blurring, and CT and MRI of the brain showed no thrombus or infarcted area. Brain CT angiogram along with negative brain imaging leaves an MLF lesion unlikely. Myasthenia gravis, GBS, or the Miller-Fisher variant of GBS being the commonest etiologies associated, the possibility of a pseudo-INO needs to be considered [12]. There was no background history to suggest GBS and no history of ataxia or muscle weakness. Although the RNS test is negative, AChR antibody positivity and remarkable improvement to acetylcholinesterase inhibitors directed us toward ocular myasthenia gravis.

Management of MG requires acetylcholinesterase inhibitor pyridostigmine. Glucocorticoids and other immunomodulatory drugs have a place in those who are symptomatic while on acetylcholinesterase inhibitors. Pulsed steroids, intravenous immunoglobulin, and plasma exchange are reserved for emergencies such as myasthenic crises. The prognosis could be variable. Old age, a history of myasthenic crisis, inadequate symptom control, and the presence of a thymoma carry a poor prognosis [13].

## Conclusions

Pseudo-INO is a rare presentation of MG. Since there are numerous atypical presentations of MG and many mimickers for MG despite the early onset, the diagnosis and treatment initiation could be delayed. When presented with an INO excluding an MLF lesion, pseudo-INO should be taken into consideration. Prompt diagnosis and initiation of treatment will help achieve symptom control and minimize myasthenic emergencies.
